# Managing carbon waste in a decarbonized industry: Assessing the potential of concrete mixing storage

**DOI:** 10.1007/s11356-023-31712-0

**Published:** 2024-01-05

**Authors:** Vitor Sousa, Rita Nogueira, Inês Meireles, André Silva

**Affiliations:** 1 CERIS, Department of Civil Engineering, Architecture and Georesources, Instituto Superior Técnico, University of Lisbon, Av. Rovisco Pais, 1049-001 Lisbon, Portugal; 2 RISCO, Department of Civil Engineering, University of Aveiro, Campus de Santiago, 3810-193 Aveiro, Portugal; 3 Department of Civil Engineering, Architecture and Georesources, Instituto Superior Técnico, University of Lisbon, Av. Rovisco Pais, 1049-001 Lisbon, Portugal

**Keywords:** Concrete production, Carbon dioxide emissions, Carbon storage, Concrete mixing

## Abstract

The effort towards a greener future will entail a shift to more environmentally friendly alternatives of many human activities. Within this context, the path towards a decarbonized society in general, and industrial decarbonization in particular, will require using low carbon solutions and/or capturing carbon emissions at the source. This flux of captured carbon will then require management and one option is to store it in concrete. The incorporation of the captured CO2 can be done during the mixing and/or curing. While the latter is more efficient and effective in terms of the amount of CO2 incorporated, it is limited to concrete in elements that are compatible with chamber curing. In practice, this would be restricted to the concrete pre-fabrication industry and, most probably, only to small size elements. Despite the lower performance, incorporation of CO2 into concrete during the mixing stage is a relatively universal alternative. The present research effort reveals that the latter solution is beneficial from an environmental point of view, with an estimated yearly carbon storage of 23 million tonnes worldwide against emissions of 2.5 million tonnes to do it.

## Introduction

Greenhouse effect is essential to make life, as we know it, possible in the planet by retaining a portion of the solar energy in the atmosphere. According to Manabe (2019), greenhouse gases (GHG) are responsible for the temperature difference of roughly 33ºC between the measured mean air temperature (14.5ºC) and the theoretical estimated temperature if the Earth-atmosphere system would behave like a Stefan–Boltzmann blackbody (-18.7ºC). The same author also explains how the GHG concentration increase, in particular carbon dioxide (CO2) since it is the most abundant from anthropogenic origin (IPCC 2014), leads to an increase of the planet mean air temperature. The temperature increase causes also an acceleration of the water cycle, resulting in a higher average water vapor concentration in the atmosphere. Since water vapor is the most abundant GHG in the atmosphere and is responsible for half of the greenhouse effect (Buis 2022), this further exacerbates climate changes.

Considering Earth as a closed system (except for the energy exchanges to and from space), the issue associated with anthropogenic GHG emissions is that it represents net transfer of substances with greenhouse effect potential, presently stored in the solid or liquid forms in the surface or the crust, to the atmosphere, in the gaseous state. Basically, the problem is the destabilization of the natural balance between emission and removal of GHG to and from the atmosphere that human activities are responsible for. Amongst those activities, the built assets assume a spotlight position in terms of GHG emissions, with the operation of buildings being responsible for 28% of the anthropogenic carbon dioxide emissions (CO2) emissions worldwide, and further 11% being attributed to the construction industry (IEA 2019).

Within the construction industry, the largest portion of the CO2 emissions are from the production of the construction materials. In this context, structural materials such as concrete and steel are responsible for a significant portion since they are present in buildings and infrastructures alike, while other constructions materials (e.g., glass) are only present in buildings. Steel (160 kg CO2/t for recycled rebar to 6150 kg CO2/t for stainless steel) has a substantially higher specific embodied carbon than concrete (72 kg CO2/t for concrete with 75% of ground granulated blast slag to 375 kg CO2/t for autoclaved aerated blocks) (Cabeza et al. 2021). However, concrete is not only the most consumed construction material, but it is the most consumed material overall worldwide after water (WBCSD 2009; ISO/TC 071 2016). Considering the yearly concrete production of 30 billion tones reported by Monteiro et al. (2017) and assuming that concrete is responsible for 9–10% of the global CO2 emissions indicated by Cao et al. (2021), an average emission of 120 kg CO2/t is obtained. This figure is slightly higher than the 72.5 kg/t indicated by MPA (2021), but in the lower end of the range of values of the studies reviewed by Cabeza et al. (2021).

Concrete is a mixture of three basic components, aggregates, cement and water, but it may include also other additives and/or admixtures, usually in minor proportions. With an estimated consumption of 2.4 tones per cubic meter of concrete, aggregates make up roughly 60% to 75% of the concrete volume (Wang et al. 2021; Warburton 2020). However, with GHG emissions ranging between 7.85 and 103 kg CO2eq/t, aggregates imply mostly the consumption of large amounts of natural raw material (Hossain et al. 2016; Bascetin et al. 2017). On the other hand, cement comprise 15% to 20% of the concrete volume but is responsible for 75% of the concrete CO2 emissions (Cao et al. 2021). With specific carbon emissions usually above 600 kg CO2/t, for blended cements, and above 800 kg CO2/t, for non-blended cements (Anderson and Moncaster 2020), Portland cement is responsible for 7–8% of all anthropogenic CO2 emissions (Miller et al. 2016; IEA 2018; Andrew 2018) and concrete incorporates the largest portion of the over 4 billion tones produced annually worldwide (USGS 2022).

While some sources of carbon emissions are possible to replace by carbon neutral alternatives (e.g., renewable energies for electricity production), others are not. Portland cement in particular falls in the latter category since the emissions from the production of its main component, the clinker, originate from two sources: i) production emissions; and ii) process emissions. The production emissions refer to the emissions associated with thermal and electrical energy consumption. The sintering reactions responsible for the formation of the clinker require maintaining the raw material at 1450ºC, which is achieved by the combustion of a variety of fossil fuel mixes. Consequently, the thermal energy required is the major source of production emissions. The presence of carbonates in the raw material, in particular calcium carbonate, is responsible for the process emissions. When the clinker raw material reaches the 800ºC-900ºC, the CO2 in the carbonates is released into the atmosphere. Process emissions make up around 60% of the total CO2 emissions from cement production (almost 70% in the most efficient cement factories) (IEA 2018; Carriço et al. 2020; Fennell et al. 2021), corresponding to around 520 kg CO2/t of clinker (IPCC 2006). So, even if it would be possible to eliminate completely the consumption of fossil fuels and use green electricity in the production of Portland cement, the calcination of the carbonates in the raw material would still be responsible for the emission of substantial amounts of CO2. This is why Davis et al. (2018) categorizes cement as one of the main energy services and industrial process that are particularly hard to provide without emission of CO2, along with long-distance freight transport, air travel, highly reliable electricity, and steel manufacturing.

Over the years, a multiplicity of alternatives has been explored to reduce the CO2 emissions from cement production by both the scientific and technical communities. These can be grouped into alternatives aiming at (Barcelo et al. 2014; Carriço et al. 2020; Gartner and Hirao 2015): i) increasing energy efficiency of the production process; ii) reducing the specific emissions by using alternative fuels (e.g., biomass, wastes) or decarbonated raw materials (e.g., chemical decarbonation); iii) incorporating alternative additives, both inert (e.g., limestone filler) or active (e.g., fly ash), to reduce the clinker content of the cement or concrete; and iv) capturing and storing CO2 in the gases before releasing to the atmosphere. Several solutions within the first three groups are already used by the industry for years, but their mitigation potential has been eliminated in terms of overall net emissions by the exponential growth of the demand for cement, which is reported to have increased by a factor of 30 since 1950 and almost 4 times since 1990 (Andrew 2019). As a result, the proportion of the global anthropogenic CO2 emissions resulting from cement production is estimated to have increased from 5%, in 2000, (Worrell et al. 2001) to 8%, in 2018 (Andrew 2018). The evolution of the total CO2 emissions from just over 25 to almost 35 billion metric tons in the same period (Friedlingstein et al. 2022) means that the increase is even larger in absolute terms.

In order to achieve carbon neutrality in the cement industry, in particular, and the construction industry, in general, the development and implementation of more efficient CO2 capture technologies will be required (Wilberforce et al. 2021). According to the CEMBUREAU (2020) and the PCA (2021) roadmaps, carbon capture technologies will be responsible for up to 42% of the CO2 emissions reduction needed for carbon neutrality of cement. This, in turn, will generate a significant amount of CO2 waste that will need to be managed. Some management alternatives that are being assessed include (Hepburn et al. 2019): i) storage in geological formations (e.g., empty gas fields); ii) storage in biomass (e.g., algae); iii) use in the production of chemicals (e.g., urea) and fuels (e.g., methanol); iv) use in forestry and agricultural applications (e.g., soil organic carbon content); and v) incorporation in materials (e.g., concrete).

Looking at the last option, concrete (mostly the cement) is not just a source of CO2 emissions. The natural carbonation reaction between CO2 and the hydrated cements compounds, makes it also a sink for atmospheric CO2. Cao et al. (2020) analysis of the cement carbon cycle estimated that, in 2014, cement production was responsible for emitting almost 3 Gt of CO2 but, at the same time, absorbed more than 0.6 Gt of CO2. Almost 80% of the CO2 uptake from cement-based products (mortar and concrete) takes place during the life cycle of the built assets. In addition to the fact that the balance between emissions and absorptions is extremely positive, there is also a substantial time delay between emission and absorption. Considering that typical life spans of built assets range from 50 to 100 years, even if the absorption capacity was the same of the emissions there would be a substantial accumulation of CO2 in the atmosphere. Furthermore, the full absorption potential is not used, since the 50 years carbonation depths estimated by Elgalhud et al. (2017) are always below 80 mm. Pade and Guimaraes (2007) estimated that only 25% of the CO2 that could be uptaken by concrete carbonation is absorbed after 100 years (70 years of service life and 30 years in landfill as crushed concrete).

Assuming that no alternative materials are developed for replacing concrete and Portland cement in the near future and that carbon capture will be required to meet the emission targets, CO2 will become a substantial by-product flux from the cement industry. A possibility for dealing with it is to accelerate its absorption by concrete during the mixing and/or curing stages. Herein, we assess the balance between the CO2 stored and the emitted for injecting CO2 during concrete mixing, in particular in the ready-mix industry, but also the precast industry. Assuming that CO2 capture will be mandatory in cement plants in the near future, the CO2 emitted comprise the liquefaction, transportation, vaporization and injection stages required for injecting CO2 during concrete mixing.

The potential of incorporating CO2 into concrete is not limited to the acceleration of the natural carbonation reaction in conventional concrete (e.g., Meng et al. 2022). Another alternative based on the same principle is the accelerated carbonation of recycled aggregates (e.g., Singh et al. 2021; Kursula et al. 2022; Torrenti et al. 2022; Zhang et al. 2023) prior to the production of recycled aggregates concrete. However, a variety of other options have explored using other concrete components as CO2 absorbers. One to these options is the production of nano-calcite to be used as filler in concrete (e.g., Qin et al. 2018; Fu et al. 2022; Liu et al. 2022; Monkman et al. 2022). Batuecas et al. (2021) demonstrates that is possible to use waste CaO, for instance from the purification of steelmaking slags or from the by-product of the soda ash Solvay production process (calcium chloride and NH3), as source of calcium to obtain the nano-calcite by mixing mix CO2. The carbonation of alkaline mineral concrete admixtures, such as fly ash or steel slag, has been also explored as an alternative to incorporate CO2 into concrete (e.g., Pan et al. 2017; Humbert and Castro-Gomes 2019). Presently, there is already a variety of technologies aimed at carbonizing various types of wastes to produce artificial aggregates (e.g., Hanifa et al. 2023).

## Injecting co2 during concrete mixing 

The artificial storage of CO2 in concrete can be done by either (Nogueira et al. 2023): i) curing the concrete in CO2 rich atmospheres; and ii) mixing concrete with CO2. The former is, in practical terms, limited to concrete used in the precast industry, since it requires placing the elements in an air-tight chamber to enable the creation of the CO2 rich environment. This might even be limited to precast concrete elements of small size. Injecting CO2 during concrete mixing consists in using CO2 as component during the production stage. This approach can be easily expanded to all ready-mix concrete industry and even to on-site concrete batching plants, such the ones that can be found in large construction projects (e.g., dams).

When injecting CO2 during concrete mixing, the typical reactive compounds of concrete (cement, water and eventual additives and admixtures) are mixed with CO2 and the inert components (aggregates) at the same time. As such, the chemical reactions taking place are not the carbonation of hydrated cement compounds, since they are not formed yet. The processes occurring within this mixture are not yet fully understood, since the research is still quite recent. A few research groups are exploring this solution and some performance reductions have been observed. Table 1 resumes some main results obtained with injecting CO2 during concrete mixing and the overall conclusion is that the amount of CO2 injected has to be lower than 1% of the cement weight to prevent the degradation of the concrete performance. The exception is the recent work by Liu et al. (2021), but the authors do not discuss or compare the results with previous research efforts in the topic.

**Table 1:** Summary of results from carbonation of cement-based materials during mixing

Material	Carbonation process	Performance	Footprint benefit	Refs
Concrete (308 kg of cement and 77 kg of slag)	0.05%wt 0.15%wt 0.30%wt (CO_2_/cement) injected during mixing	3% increment 4% reduction 6% reduction In *fc*^1^ at 28 days No impact on durability	not mentioned	Monkman and Cail (2019)
Concrete (147.7 kg of cement and 73.9 kg of slag and of fly ash)	0.11%wt (CO2/cement) injected during mixing	No reduction in *fc*^1^ in relation to a reference concrete with 4.3% more binder	net reduction in CO_2_ of 10.0 kg/m^3^ of concrete	Kwasny et al. (2014)
Cement paste (w/c = 0.5, 0.6 and 0.7)	0.44%wt 1.76%wt 0.88%wt 1.32%wt 2.20%wt (CO2/cement) injected during mixing	Decrease Increase Increase Increase Increase In *fc*^1^ at 28 days (graphical data)	not mentioned	Liu et al. (2021)
Cement paste (w/c = 0.4)	Carbonated water with 4.2 pH (CO_2_ introduced at a pressure of 8 bar)	20% reduction In *fc*^1^ at 28 days	not mentioned	CarbonCure (2021)
Cement mortar (w/c = 0.4)	Carbonated water with 4.2 pH (CO_2_ introduced at a pressure of 8 bar)	Reduction in *fc* Porosity doubled and pores around 80 nm increased	not mentioned	CarbonCure (2021)
Cement paste (w/c = 0.5)	Carbonated water with 4.2 pH (CO_2_ introduced at a pressure of 1 bar)	6% reduction in *fc*^1^ at 28 days	not mentioned	Lippiatt and Ling (2020)
Cement paste (w/c = 0.44)	45 min 90 min of mixing time in a carbonation chamber with 85 ± 5%v/v of CO_2_ concentration	12% reduction 13% reduction In *fc*^1^ at 28 days	0.93%wt 1.12%wt CO_2_ uptake (CO_2_/clinker)	He et al. (2017)

^1^*f*_*c*_ – compressive strength

In practical terms, two main strategies have been explored to introduce CO2 during concrete mixing: i) use of carbonated water (Kwasny et al. 2014; Silva et al. 2021); and ii) inject CO2 directly to the mixture (Monkman and MacDonald 2016). Since the solubility of CO2 in water is just 0.0015 gCO2/gH2O (Dodds et al. 1956), this is the factor limiting the amount of CO2 possible to store in concrete. The injection of gaseous CO2 has been used for decades in the production of wood-cement composites to increment the product turn-over (Jorge et al. 2004) and, more recently, is being implemented by CarbonCure Technology Inc. (CarbonCure 2021) in the production of ready-mixed concrete. The injection is reported to last for just 1–2 min and introducing less than 1% of CO2 by weight of cement. In laboratory conditions, some authors (e.g., Kwasny et al. 2014) also explored mixing concrete inside a carbonation chamber to create a CO2 rich atmosphere, but this option has no practical use.

It should be noted that the CO2 injected during concrete mixing is absorbed not only by the cement, but also by other supplementary cementious materials that are frequently used. Several of the supplementary materials (e.g., fly ash; steel slag) are also capable of sequester CO2 (Monkman and MacDonald 2017; Monkman et al. 2016). Conservatively, this contribution was not account for because: i) the statistics found of the concrete compositions do not disclose the supplementary cementious materials type and amount; ii) the availability of the most common supplementary materials (fly ash and steel slag) are decreasing with the trend for closing coal power plants and replacing steel production from raw material by steel recycling; and iii) recent research efforts (e.g., Luo et al. 2022) are consistent with the initial studies that the CO2 absorption of the supplementary cementious materials (e.g., Kwasny et al. 2014) and the impacts on the concrete properties are identical or even better than with concrete only with Portland cement.

## Methods

### Scope

The present research was developed in the premise that, to meet the carbon emission targets needed to halt climate changes, CO2 will have to be captured in the cement and other industries. These will create the opportunity to use the CO2 captured as a non-fossil carbon-source to produce products that cannot be made without carbon and for which no non-carbon alternatives exist (e.g., many chemicals, solvents, fuels, detergents) (Sick et al. 2022). However, the amount of CO2 that is estimated to be required to capture exceeds the demand by these products. So, before considering its storage as a waste, it is considered herein that there are economic and environmental benefits from incorporation CO2 in products that currently are not made with carbon. In this context, the concrete industry has a large potential by allowing the CO2 to be used for carbonating aggregates and mixing and curing concrete (Woodall et al. 2019).

**Fig. 1:**
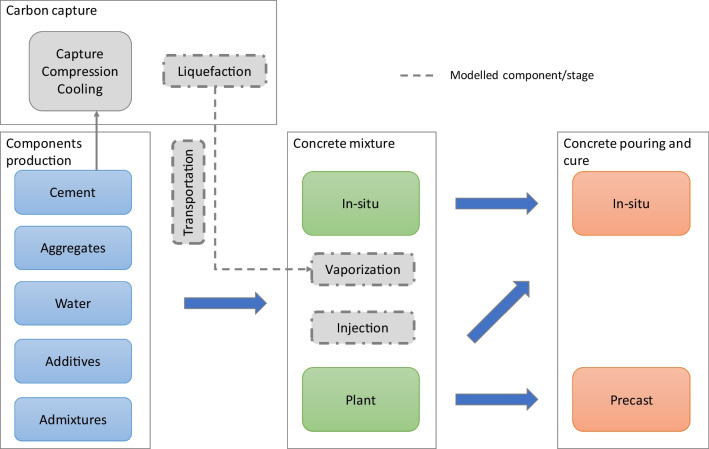
Modelled system boundaries

Within this context, the evaluation of the CO2 balance of injecting CO2 during concrete mixing in the concrete industry can be modelled considering the system defined in dashed lines in Fig. 1. Only the stages that are different from the traditional concrete production stage and required to inject the CO2 into the concrete during the mixture are modelled, which include the liquefaction, the transportation, the vaporization and the injection of the CO2. The functional unit for reference the modelling is 1 m^3^ of concrete produced.

The exclusion of the capture compression and cooling of the CO2 from the modelled system boundaries results from the assumption that carbon capture will become mandatory in the near future. In this circumstance, the energy consumption in these stages will be associated with the production process that emits it. This implies the assumption that the CO2 will be captured at the cement factories, creating a product loop aligned with the circular economy model reflected in the European Green Deal (EU 2020). This approach has similarities with the environmental performance assessment of several subproducts that are incorporated into concrete for several decades, in particular fly ash (Xu and Shi 2018).

### Methodology

The stages identified in dashed in Fig. 1 were simulated to determine the balance between the CO2 storage and emitted with injecting CO2 during concrete mixing. If the balance is positive, the amount of CO2 stored in the concrete is less than the CO2 emitted in the process, rendering it an environmentally viable option.

Fundamentally, the amount of CO2 that can be stored by injecting CO2 during concrete mixing is the product between the: i) absorption rate; ii) the amount of concrete produced; and iii) the cement content of the concrete. The absorption rates translate the amount of CO2 that is retained in the concrete. The amount of concrete produced was limited to ready-mix and precast industries, since injecting CO2 during concrete mixing on construction sites may be difficult in some cases. Since the absorption rate is by weight of cement, knowing the average cement dosage or total cement consumption in ready-mix and precast concrete is required.

**Fig. 2:**
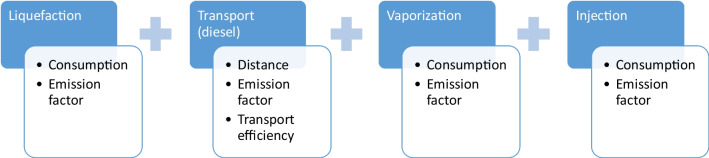
Stages emission variables in the CO_2_ supply chain

The emissions from implementing carbon curing can be estimated by the product between the emission factor and the energy consumption in each stage, as represented in Fig. 2. With the exception of the transportation, all other processes are assumed to consume electricity, so the emission factor will vary between country depending on the mix of energy sources. The electricity consumption depends on the equipment required for each stage. The transportation emissions depend on the distance, the emission factor and the transport efficiency. The distance was estimated as the diagonal of the square corresponding to the area of each country divided by the number of cement factories. The emission factor and transport efficiency (reflects the weight of the container in relation to the product being transported) were estimated assuming road transportation using trucks.

Given the degree of uncertainty in all the model elements, a stochastic analysis was also carried out resorting to Monte Carlo simulation, as a complement to the deterministic approach.

### Data

A mixed approach was adopted to obtain the data required to run the simulations, including: i) industry associations and official sources (CO2 emissions from electricity generation and land transportation; concrete production; ready mix production; precast production; cement content); ii) research results from the literature (concrete production; CO2 absorption; precast production; cement content; energy consumption); and iii) questionnaire replies (precast concrete consumption and composition). The questionnaires were just conducted in Portugal and only six complete replies were obtained, representing roughly 5% of the market.

The absorption rates considered herein were retrieved from Ravikumar et al. (2021). The absorption rates reported by Ravikumar et al. (2021) range between 0.001 kg CO2/kg cement and 0.016 kg CO2/kg cement, having an average value of 0.0085 kg CO2/kg. The 0.01 kg CO2/kg cement limit for avoiding performance degradation of the concrete was taken into consideration, but assuming that in some cases it could be slightly exceeded in the stochastic modeling.

Table 2 lists the information collected in terms of total concrete production and the amount used in the form of ready-mix concrete and precast elements. The distribution of the total concrete consumption in 2020 was estimated assuming the same proportion distribution presented by Miller et al. (2016) and using the total concrete production indicated by the GCCA (2021). The data regarding the concrete used in the precast industry is scarcer and it was impossible to obtain with the same temporal discretization.

**Table 2:** Concrete statistics by location and use (Miller et al. 2016; ERMCO 2017, 2020; GCCA 2021; Sick et al. 2022; Statistica 2022; Vázquez-Calle et al. 2022; Wang et al. 2021)

Region	Concrete [10^6^ m^3^]							
	Total		Ready mix					Precast
	2 020^a^	2 016	2 020	2 019	2 018	2 017	2 016	Various
Africa	628.7	451.7						
Nigeria			63.9					
America								
Brazil	246.9	177.4	115.0					
Colombia			5.3					
Canada	39.4	28.3						
United States	353.4	253.9	286.0	280.0	274.0	270.0	265.0	25.2
Rest of America	367.6	264.1						
Europe	849.6	610.4						
Turkey			95.0	67.0	100.0	115.0	109.0	
Germany			55.3	53.5	52.8	51.7	49.5	
France			37.0	40.4	40.1	38.8	36.3	12.2^b^
Italy			28.7	28.4	27.3	27.3	23.3	
Poland			25.7	26.2	25.1	20.4	20.4	
United Kingdom			24.9	24.9	22.5	22.9	24.6	
Spain			22.8	24.8	22.2	16.3	16.3	
Belgium			12.4	13.0	12.8	12.7	12.5	
Austria			11.5	11.9	11.8	11.0	10.8	
Switzerland			11.4	11.1	10.9	11.5	11.5	
Netherlands			7.4	7.8	7.5	6.9	6.5	
Portugal			5.9	5.1	4.5	3.5	3.5	3.4^c^
Denmark				2.7	2.6	2.6	2.5	
Sweden				4.5	4.5	4.5	4.5	
Ireland				4.3	4.3	4.3	2.4	
Slovakia				2.8	3.0	2.4	1.9	
Norway				3.8	4.1	4.1	4.0	
Czechia				7.1	7.1	6.8	6.8	
Finland				2.7	2.8	3.0	2.9	
Middle East	719.2	516.7						
Iran			141.0					
Israel				18.9	18.0	16.9	15.4	
Asia								
China	7 940.4	5 704.7	2 848.0				1 200^**d**^	
India	1 027.0	737.8	537.0					
Japan	175.8	126.3	89.2	84.8	84.8	84.0	99.0	
Indonesia			119.0					
Pakistan			91.4					
Thailand			2.6					
Taiwan			42.1					
Russia				38.0	38.0	35.0	37.0	
CIS	406.4	292.0						
Oceania								
Australia and New Zealand	46.4	33.3						
Rest of Asia and Oceania	1 199.1	861.5						
**World**	**14 000**	**10 058**					**2 400**^**d**^	**4 200**

^a^ estimated assuming the same proportional distribution ofMiller et al. (2016)

^b^ estimated from the precast weight considering an average specific weight of 1.7 t/m3 derived from world total precast products weight of 7000 × 10^6^ t (Sick et al. 2022)

^c^ estimated from replies to industry questionnaire on precast concrete cement dosage and extrapolation based on cement consumed on the precast industry

^d^ value estimated for 2015 by Vázquez-Calle et al. (2022), considered conservative since the share of ready-mix in China increased from 32 to 38% between 2008 and 2010 and was estimated to increase to 51% by 2013 (Song 2011)

For some countries, the data in Table 2 allows estimating the proportion of concrete that is used in the form of ready-mix concrete (Brazil: 46.6%; United States: 80.9%; China: 35.9; India: 52.3%; Japan: 50.7%). The average of the proportion of concrete used as ready mix weighted by the amount of concrete consumed in each country is 39.8%. Assuming this percentage to be representative of the world, the amount of ready-mix concrete produced in 2020 can be estimated to be 5 568.1 × 10^6^ m^3^.

The cement content of the ready-mix concrete is presented in Table 3, along with the estimate of the transportation distances. The 2020 estimate of cement content was based on the APEB (Associação Portuguesa das Empresas de Betão Pronto) estimate that 41% of the total of 4 × 10^6^ t of cement consumed in Portugal was used in ready-mix concrete. This information was shared in a meeting and is not available in an official publication. The average distances were estimated dividing each country land area by the corresponding number of cement factories and assuming that the distance corresponds to the diagonal of a square with the area per plant obtained. In most cases, the estimates are within the 200 km to 300 km limit for economic land transportation distances indicated by CEMBUREAU (https://cembureau.eu/about-our-industry/key-facts-figures/). Noticeable exceptions include Canada, Norway, Sweden and Russia, all relatively large countries with small population densities. This reveals that, most probably, the results are conservative since the installations (cement factories, ready-mix plants and prefabrication units) will tend to concentrate close to the consumption points.

**Table 3:** Average cement content of ready-mix concrete and average distance between cement factories and ready-mix plants of precast industries (ERMCO 2017, 2020; WB 2022; CEMNET 2022; USGS 2021)

Region	Cement content in ready-mix concrete					Distance
	[kg/m^3^]					[km]
	2020	2019	2018	2017	2016	2020
Africa						
Nigeria						426.8
America						
Brazil						421.7
Colombia						317.6
Canada						1058.6
United States	261	270	270	270	270	417.4
Rest of America						
Europe						
Austria		260	260	260	260	122.5
Belgium		310	280	280	280	77.8
Czechia		298	298	272	272	160.4
Denmark		260	260	260	260	200.0
Finland		355	350	350	345	450.1
France		298	297	297	298	152.6
Germany		303	305	301	298	118.2
Ireland		260	260	260	255	151.5
Italy		320	320	320	320	99.3
Netherlands		314	315	315	313	149.8
Poland		278	285	270	270	217.0
Portugal	278	244	204	195	196	151.3
Slovakia		303	300	302	300	138.7
Spain		270	275	275	283	154.2
Sweden						638.2
United Kingdom		278	258	262	260	168.7
EU		288	285	283	282	128.8
Norway		360	360	360	365	603.6
Switzerland		280	280	280	280	114.8
Turkey		305	295	290	290	142.3
Middle East						
Iran						
Israel						120.1
Asia						
China						151.8
India						143.2
Japan		348	348	351	351	153.3
Indonesia						306.4
Pakistan						234.7
Thailand						252.7
Taiwan						
Russia		360	360	359	360	699.2
CIS						
Oceania						
Australia and New Zealand						
Rest of Asia and Oceania						
**World**		**300.6**	**298.6**	**296.7**	**296.9**	**288.8**

According to the NPCA (2014), in 2013, the precast industry consumed almost 10 × 10^6^ t of cement to produce 25.2 × 10^6^ m^3^, implying an average consumption of 396.4 kg/m^3^. This value is consistent with the most recent estimates that the precast industry consumes 10% to 11% of the total cement in the US, which corresponded to an amount of 10.3 t to 11.8 t (USGS 2020, 2021, 2022). In Portugal, the APEB estimates that, in 2020, 24% of the total 4 × 10^6^ t of cement consumed in Portugal was used in the precast industry, which corresponds to an average cement content of 280.8 kg/m^3^. In France, the precast elements production in 2007 consumed 4 × 10^6^ t of cement, which corresponds to dosage of 329.1 kg/m3 (http://www.planete-tp.com/ciment-chiffres-cles-a760.html). The precast production was divided in (FIB 2020): i) 50% small building construction elements, mostly concrete blocks; ii) 20% large building precast elements, such as stairs and walls; and iii) 30% precast elements for environmental works, such as pipes. T

The electricity consumption for CO2 liquefaction, vaporization and injection were estimated from Ravikumar et al (2021), Erik et al. (2016) and Monkman and MacDonald (2017), and their mean values are 0.10 kWh/kg CO2, 0.047 and 0.037, respectively. Table 4 lists the specific emission factors from electricity generation by location.

**Table 4:** Average annual electricity emissions per kWh of electricity produced (Our World in Data 2022)

Region	Electricity emissions [g CO2/kWh]				
	2020	2019	2018	2017	2016
Africa	442.72	468.15	470.73	479.83	483.03
Nigeria	395.24	398.36	397.91	386.70	378.01
America					
Brazil	113.21	122.27	123.60	139.36	134.29
Colombia	192.63	172.38	163.30	153.38	223.11
Canada	116.97	130.01	129.47	135.87	141.48
United States	348.54	370.25	388.02	392.52	403.69
Rest of America					
Europe	264.54	285.81	301.95	313.30	316.79
Turkey	409.58	410.14	463.05	461.25	447.87
Germany	313.78	345.94	406.14	414.48	445.82
France	57.29	56.85	59.23	70.40	61.13
Italy	221.01	234.81	248.53	263.66	262.28
Poland	725.47	743.95	782.17	780.22	786.15
United Kingdom	242.15	264.39	280.62	294.30	322.79
Spain	174.66	213.08	276.92	304.75	265.81
Belgium	178.99	170.87	213.23	184.72	175.22
Austria	82.68	94.30	102.01	98.14	87.57
Switzerland	54.52	52.56	54.13	59.52	59.48
Netherlands	323.75	387.31	438.20	460.51	486.24
Portugal	207.34	263.38	301.81	370.16	298.62
Denmark	104.41	135.49	197.56	193.41	229.24
Sweden	12.21	11.87	12.24	12.18	12.81
Ireland	278.72	323.00	353.26	388.72	427.38
Slovakia	138.86	140.84	148.95	145.02	147.80
Norway	30.80	34.33	34.27	34.34	34.30
Czechia	405.22	436.84	465.91	471.06	504.19
Finland	57.06	86.30	112.54	87.64	100.45
Middle East					
Iran	440.40	488.77	476.36	451.23	457.53
Israel	527.77	531.44	539.72	544.07	544.35
Asia	526.03	538.07	546.70	550.72	556.71
China	545.89	556.29	570.10	574.97	580.14
India	624.37	630.10	655.28	658.13	665.39
Japan	426.12	450.45	448.68	482.91	503.63
Indonesia	624.68	625.29	618.91	649.26	640.62
Pakistan	294.18	313.72	286.38	345.58	371.84
Thailand	507.13	480.96	489.26	503.34	521.11
Taiwan	563.69	564.32	549.49	559.43	537.24
Russia	329.08	351.95	354.78	353.93	355.29
CIS					
Oceania	480.97	511.53	531.23	539.64	548.45
Australia and New Zealand					
Rest of Asia and Oceania					
World	**421.85**	**435.21**	**443.93**	**447.11**	**450.76**

**Fig. 3:**
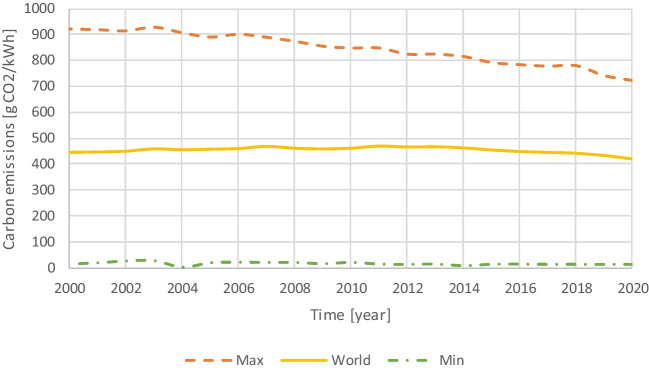
Specific electricity emission factor for the world and maximum and minimum for the countries listed in Table 2, 3 and 4

Analyzing a longer time frame (Fig. 3), it is possible to observe the overall trend in terms of specific emissions from electricity production. The highest value, which correspond to Poland, show a clear decreasing trend, but the lowest (Sweden) has been stable. At the world scale, the improvements in some of the most developed countries (e.g., Europe decreased from 360 g CO2/kWh, in 2000, to 264 g CO2/kWh, in 2020; United States decreased from 499 g CO2/kWh, in 2000, to 348 g CO2/kWh, in 2020) are being compensated by the increase in electricity demand in other regions with specific emissions higher than the average (e.g., China: 545 g CO2/kWh in 2020; India: 634 g CO2/kWh in 2020).

**Fig. 4:**
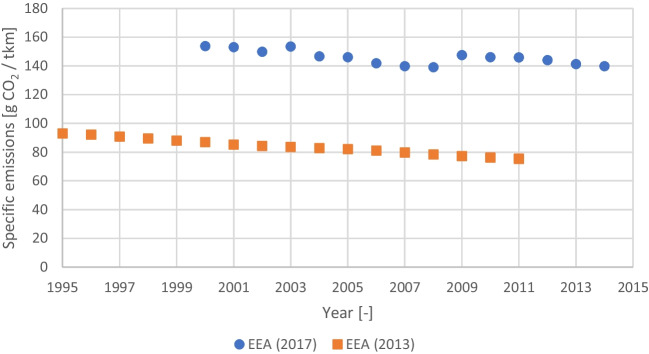
Specific CO2 emissions in road freight transportation in Europe (EEA 2013, 2017)

Freight transport emissions vary significantly with the means of transportation and the methodology used in the estimation (Wild 2021). The emission factors are, most frequently, reported not only by unit of distance (e.g., per km) but also by unit of weight (e.g., per t). Figure 4 presents two series of values reported by the EEA, enabling to grasp both the variability and order of magnitude in Europe.

From Fig. 4 it is possible to conclude that the variability over time is relatively small. The variables that have more influence on the emissions of road transportation are: i) the cargo capacity of the truck; ii) the loading ratio, or load factor; and iii) the fraction of time operating empty. Typically, the higher the capacity of the truck the lower the specific emission factor since the ratio between the weight of the cargo and of the truck diminishes (Transport and Environment 2021). The load factor is the ratio between the average effective load transported and the full capacity, reflecting how loaded is the truck usually operated. The specific emission factor tends to lower with the increase of the load factor, since it is a more efficient use of the available transportation capacity. The fraction of time operating empty could be translated in the average load factor lowering its value, implying an increase of the emissions factor (McKinnon and Piecyk (2010). The median of the average specific emissions factor values reported in McKinnon and Piecyk (2010), Transport and Environment (2021), IEA and UIC (2017), EEA (2017), Fraunhofer (2020) and Ravikumar et al. (2021) is 82 g CO2/t.km and was used in the simulations. The values reported range between less than 40 g CO2/t.km to over 700 g CO2/t.km considering the full range of heavy-duty vehicles. Considering that is more probable to transport CO2 to the ready-mix concrete plants and precast concrete factories, using medium and large heavy-duty vehicles, the maximum specific emissions is around 300 g CO2/t.km (Sims and Shaeffer 2014).

### Formulation

All input data for the Monte Carlo simulation was assumed to follow a PERT distribution (PERT). Since it is a robust measure central tendency, the median was used instead of the average to determine the most probable value using the range of values available between 2016 and 2020. For the data with limited information, namely the precast industry data, a ± 10% variation was assumed.

The equations of the simulation model are presented below. Since the model is stochastic, the results are represented by a distribution (Dist.), rather by a single value as in deterministic models.

- 1) CO2 balance ($${B}_{CO2}$$)
  $$Dist.\left({B}_{CO2}\right)=Dist.\left({S}_{CO2}\right)-Dist.\left({E}_{CO2}\right)$$
  where ${S}_{CO2}$ is the amount the amount of CO2 stored in concrete by injecting during the mixing; and ${E}_{CO2}$ is the amount of CO2 emitted to inject CO2 during concrete mixing.
- 2) CO2 stored in the concrete
  $$Dist.\left({S}_{CO2}\right)=PERT\left({C}_{Produced}\right)\times PERT\left({C}_{Content}\right)\times PERT\left({C}_{Absorption}\right)\times PERT\left({M}_{Efficiency}\right)$$
  Please check if all equations are presented correctly. where ${C}_{Produced}$ is the amount of concrete produced (Table 2); ${C}_{Content}$ is the cement dosage of the concrete produced (Table 3); ${C}_{Absorption}$ is the CO2 absorption (from Ravikumar et al. (2021)); and ${M}_{Efficiency}$ is the mixing efficiency. The latter intents to reflect that, in real conditions, some of the CO2 injected may be lost to the atmosphere (assumed a value between 0.5 and 0.85, with a median of 0.675). This is a conservative approach, since if the mixing efficiency is closer to 1 the CO2 stored will be higher for the same CO2 emitted in the process.
- 3) CO2 emitted for injecting CO2 during concrete mixing (${E}_{CO2}$) is comprised by the emissions from liquefying (${E}_{Liquifaction}$), transporting (${E}_{Transportation}$), vaporizing (${E}_{Vaporization}$) and injecting (${E}_{Injection}$) the CO2 captured at the cement plants (Fig. 1)
  $$Dist.\left({E}_{CO2}\right)=Dist.\left({E}_{Liquifaction}\right)+Dist.\left({E}_{Transportation}\right)+Dist.\left({E}_{Vaporization}\right)+Dist.\left({E}_{Injection}\right)$$
  with
  $$Dist.\left({E}_{Liquifaction}\right)=PERT\left({ED}_{Liquifaction}\right)\times PERT\left({E}_{Electricity}\right)$$
  $$Dist.\left({E}_{Transportation}\right)=PERT\left({D}_{Plants}\right)\times PERT\left({E}_{Freight}\right)\times PERT\left({T}_{Efficiency}\right)$$
  $$Dist.\left({E}_{Vaporization}\right)=PERT\left({ED}_{Vaporization}\right)\times PERT\left({E}_{Electricity}\right)$$
  $$Dist.\left({E}_{Injection}\right)=PERT\left({ED}_{Injection}\right)\times PERT\left({E}_{Electricity}\right)$$
  where ${ED}_{Liquifaction}$, ${ED}_{Vaporization}$ and ${ED}_{Vaporization}$ are the electricity demands for liquifying, vaporizing and injecting the CO2; ${E}_{Electricity}$ is the specific electricity CO2 emission factor (Fig. 3); ${D}_{Plants}$ is the distance between the cement and the ready-mix or precast plants (Table 3); ${E}_{Freight}$ is the specific CO2 emissions in road freight transportation (Fig. 4); and ${T}_{Efficiency}$ is the transportation efficiency. The latter reflects that the load transported is no all CO2 since there is also the CO2 containers and the trucks do not return fully load from the ready-mix and precast plants (assumed a value between 0.585 and 0.715, with a median of 0.65).
  The full data used in all stages for modeling the results presented below is detailed in Table 5 of the appendix.

## Results and discussion

**Fig. 5:**
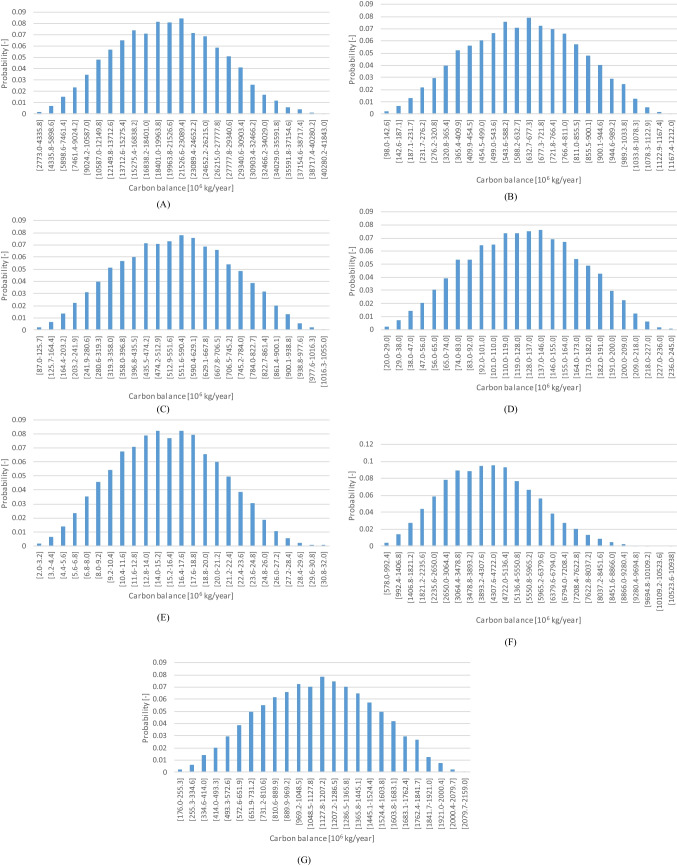
Balance between the CO2 stored and emitted during carbon curing in: (**A**) World; (**B**) United States; (**C**) European Union; (**D**) France; (**E**) Portugal; (**F**) China; and (**G**) India

Figure 5 presents the results of the balance (difference) between the amount of CO2 that is possible to store and the CO2 that emitted with injecting CO2 during the mixture of concrete for the World, US, European Union, France, Portugal, China and India. The results were present only for these locations because: i) there is a lack of complete data for all locations listed in Table 2, 3 and 4; ii) the results are similar (the graphs in Fig. 5 are similar, with just a shift in the order of magnitude), so it is possible to avoid excessive repetition; iii) the major concrete and cement producers are captured; and iv) the difference between very large (US, China), large (India), medium (France) and small countries (Portugal) is captured.

The results are limited to the concrete produced as ready-mix concrete, except for the World, US, France and Portugal, that also include the cementious elements produced in the precast industry.

The sensibility analysis indicates that, in most cases, more than 95% of the variability is dictated by the CO2 absorption during the mixing. The only exception is China, where the ready-mix concrete consumption is responsible for 15% of the variability, reducing the importance of the CO2 absorption to 84%. This is explained by the fact that back, in 2010, the proportion of ready-mix concrete in relation to the total concrete produced per year was only 35%, whereas in more developed countries (e.g., EU) the proportion was already above 60%. For instance, in the US the proportion of concrete produced as ready-mix has been steady between 70 and 75% for several years and in China it has been growing. Most probably, this is not observed in India because of the lack of data prior to 2020.

The balance is extremely positive, with the emissions from implementing CO2 injection during the mixing stage being roughly 11% of the amount of CO2 that is possible to store in the concrete. However, the processes developed at laboratory scale indicate CO2 absorption efficiencies ranging between 0.5 and 0.85, with an average value of 0.675. If the CO2 lost to the atmosphere during the mixing stage is accounted for as emissions, the balance is significantly worse, with an average world value of almost 60%. This is clearly a bottleneck to the solution for which there no information disclosed in the industrial implementation by CarbonCure.

## Conclusions

The present research effort aims at estimating the potential for using concrete for storing CO2 by its incorporation during the mixing stage. The simulation model developed was built in the assumption that its capture will be required for various industries to meet increasingly stringent emissions targets, in particular the cement industry. This assumption implies that the energy consumption for capturing the CO2, and corresponding emissions, are not a burden on their use in concrete.

In this context, the balance is highly positive. The world average CO2 emissions for compressing, transporting, vaporizing and injecting the captured CO2 into the concrete mixing at ready-mix concrete plants or precast concrete factories are just roughly 11% of the amount of CO2 that is possible to store into concrete. Since the CO2 emissions from injecting CO2 during concrete mixing are mostly from electricity consumption, the values vary between 2.4%, in France, to 14%, in India, due to the differences in the energy sources mix for electricity generation.

Considering that the CO2 emission from cement production amounted to 1 626 × 10^6^ t in 2020 (Global Carbon Altas 2022), injecting CO2 during concrete mixing could only provide storage for a little more than 1%. As such, this will not solve the CO2 waste management problem that implementing carbon cure in large scale will create.

## Data Availability

All data and respective sources are in the manuscript.

## Appendix

See Table 5

**Table 5:** Full simulation inputs and results

Parameter	Median	Max	Min
Electricity			
Emission factor [g CO2 / kWh]			
World	443.93	450.76	421.85
US	388.02	403.69	348.54
EU	301.95	316.79	264.54
France	59.23	70.40	56.85
Portugal	94.30	102.01	82.68
China	570.10	580.14	545.89
India	655.28	665.39	624.37
Consumption [kWh / kg CO2]			
Liquefaction	0.09	0.14	0.08
Vaporization	0.007	0.0088	0.0053
Injection	0.037	0.041	0.033
Fuel (transportation)			
Distance [km]			
World	288.79	450.14	77.82
US	417.42	450.14	77.82
EU	128.82	450.14	77.82
France	152.64	450.14	77.82
Portugal	151.33	450.14	77.82
China	151.80	450.14	77.82
India	143.19	450.14	77.82
Emission factor [g CO2 / t.km]	82.00	300.00	40.00
Efficiency [kg CO2 / kg transported]	0.650	0.715	0.585
Specific emission carbon mixing** [**kg CO2 emitted / kg CO2 used]			
World	0.074	0.183	0.052
US	0.074	0.174	0.043
EU	0.047	0.158	0.033
France	0.016	0.110	0.009
Portugal	0.021	0.116	0.012
China	0.084	0.208	0.067
India	0.095	0.225	0.076
Ready-mix concrete			
Consumption [10^6^ m^3^ / year]			
World	3984.04	5568.08	2400.00
US	274.00	286.00	265.00
EU	246.23	260.00	230.03
France	38.70	40.30	36.10
Portugal	4.50	5.90	3.20
China	2024.00	2848.00	1200.00
India	537.00	537.00	537.00
Cement content [kg / m^3^]			
World	293.55	298.68	292.22
US	270.00	270.00	270.00
EU	284.00	288.00	282.00
France	297.50	298.00	297.00
Portugal	200.00	244.00	195.00
China	292.66	298.27	291.27
India	292.66	298.27	291.27
Precast concrete			
Consumption [10^6^ m^3^ / year]			
World	4200.00	4620.00	3780.00
US	25.23	27.75	22.71
EU			
France	12.15	13.37	10.94
Portugal	3.42	3.76	3.08
China			
India			
Cement content [kg / m^3^]			
World	366.67	403.34	330.00
US	396.40	436.04	356.76
EU			
France	329.10	362.01	296.19
Portugal	280.82	308.91	252.74
China			
India			
Mixing performance			
Absorption [kg CO2 / kg cement]	0.0085	0.016	0.001
Efficiency [kg CO2 absorbed / kg CO2 mixed]	0.675	0.85	0.5
Storage [10^6^ kg / year]			
World	23,031.1	56,423.9	1948.7
US	713.8	1429.1	79.7
EU	594.4	1198.1	64.9
France	131.9	269.6	14.0
Portugal	15.8	41.6	1.4
China	5034.9	13,591.7	349.5
India	1335.8	2562.8	156.4
Emissions [10^6^ kg / year]			
World	2540.49	12,175.34	202.10
US	78.13	293.14	6.88
EU	41.42	222.14	4.31
France	3.13	34.93	0.24
Portugal	0.48	5.69	0.03
China	626.13	3331.54	46.53
India	187.65	677.70	23.73
